# Brief communication: characteristics of primary drug resistance in newly diagnosed HIV-infected individuals in Ganzhou, China

**DOI:** 10.1186/s12981-025-00758-0

**Published:** 2025-06-16

**Authors:** Dan-Dan Huang, Jun-Jie Liu, Ya-Ting Chen, Rong-Rong Yang, Jun-Zhi Su, Qian Gao, Xin Chen

**Affiliations:** 1 Department of Pathogenic Biology, School of Basic Medical Sciences, Gannan Medical University, No. 1 Hexie Avenue, Zhanggong District, Ganzhou, 341000 China; 2 Department of Laboratory, Ganzhou Center for Disease Control and Prevention, Ganzhou, 341000 China; 3 Hospital-Acquired Infection Control Department, the People’s Hospital of Xiangtan County, Xiangtan, 411100 China; 4 Department of AIDS/STD Control and Prevention, Ganzhou Center for Disease Control and Prevention, Ganzhou, 341000 China

**Keywords:** HIV, Primary drug resistance, Integrase strand transfer inhibitor, Ganzhou

## Abstract

**Background:**

Primary drug resistance (PDR) is an important cause of antiretroviral therapy (ART) failure. However, the prevalence and characteristics of PDR in Ganzhou remain unclear.

**Methods:**

From July 2018 to August 2021, treatment-naïve, newly diagnosed HIV-infected individuals in Ganzhou, China were recruited. Blood samples were collected, and the HIV *pol* gene was amplified by nested PCR followed by Sanger sequencing. Sequence editing and assembly were performed using DNASTAR Lasergene software, and subsequent analysis for resistance mutations and drug susceptibility profiling was conducted using the Stanford University HIV Drug Resistance Database.

**Results:**

Among 108 successfully amplified samples, seven exhibited low-, intermediate-, or high-level resistance mutations, resulting in a PDR prevalence of 6.5%. Among them, the mutation rate of non-nucleoside reverse transcriptase inhibitors (NNRTIs) was 4.6%, and the drug resistance mutation rates of nucleoside reverse transcriptase inhibitors and integrase strand transfer inhibitors (INSTIs) were both 0.9%. No protease inhibitor resistance was detected. Nine drug resistance mutations were detected, among which six were related to NNRTIs, one was related to nucleoside reverse transcriptase inhibitors, and two were related to INSTIs. The K103N and Y181C mutations conferred intermediate-to-high resistance to NNRTIs, while A98G and V179E caused low-to-intermediate resistance to NNRTIs, and the remaining mutations led to low drug resistance to the respective drugs.

**Conclusions:**

Compared to other regions in China, Ganzhou exhibits a relatively low PDR among newly diagnosed HIV-infected individuals. However, the emergence of INSTI-resistant strains underscores the need for enhanced resistance surveillance to prevent the spread of drug-resistant strains caused by ART failure.

## Introduction

With the global implementation of antiretroviral therapy (ART), the life expectancy of individuals infected with human immunodeficiency virus (HIV) has approached that of the general population [1]. However, the emergence of drug-resistant strains under selective drug pressure, transmitted through sexual contact and vertical transmission, has rendered virological failure a critical challenge in global HIV control [2]. According to 2024 World Health Organization data, over 10% of newly diagnosed HIV cases worldwide involve drug-resistant strains, with the extensive spread of non-nucleoside reverse transcriptase inhibitor (NNRTI)-resistant variants threatening the efficacy of efavirenz (EFV)-based first-line regimens [3].

Since China implemented the “treat-all” strategy in 2016, ART coverage has increased significantly, exceeding 90% [4, 5]. According to *China’s HIV/AIDS Diagnosis and Treatment Guidelines (2024)*, the current recommended first-line ART regimens primarily consist of two nucleoside reverse transcriptase inhibitors (NRTIs) - tenofovir disoproxil fumarate (TDF)/tenofovir alafenamide plus lamivudine (3TC)/emtricitabine - combined with a third agent from the following classes: integrase strand transfer inhibitors (INSTIs) such as dolutegravir (DTG) or raltegravir; NNRTIs including doravirine or ainuovirine; or protease inhibitors like darunavir/cobicistat. However, prolonged drug exposure and suboptimal adherence have contributed to rising drug resistance rates. National surveillance data from 2023 revealed an intermediate-level prevalence (7.8%) of transmitted drug resistance, with NNRTI resistance increasing from 1.8% (2004–2007) to 6.7% (2020–2022) [6].

Ganzhou, the largest prefecture-level city in Jiangxi Province, is a key hub connecting the Yangtze River Economic Belt and South China’s economic zone. Despite maintaining low HIV prevalence, this rapidly developing region experiences frequent population mobility and exhibits significant HIV genetic diversity, with cocirculating subtypes including CRF01_AE, CRF07_BC, CRF08_BC, and unique recombinants [7]. Our previous study of 2,204 ART-treated patients in Ganzhou found virological failure (viral load ≥ 200 copies/mL) in 12.67% of cases (*n* = 279) [8]. Without timely regimen adjustment, resistant strains from these cases may spread through high-risk behaviors, potentially increasing primary drug resistance (PDR) and undermining ART effectiveness [9, 10].

This study characterizes drug resistance among newly diagnosed HIV-infected individuals in Ganzhou, aiming to guide optimal initial treatment strategies and curb the transmission of resistant variants.

## Methods

### Study design and participants

To investigate the prevalence and characteristics of PDR in Ganzhou, treatment-naïve, newly diagnosed HIV-infected individuals from July 2018 to August 2021 were enrolled. The inclusion criteria were as follows: (1) newly diagnosed HIV infection with no ART history; (2) age ≥ 18 years; (3) Clinically stable without AIDS-defining symptoms.

### Sample collection and processing

Demographic data (gender, age, marital status, education, transmission routes) were recorded. Five milliliters of peripheral blood were collected in EDTA-anticoagulated vacuum tubes and centrifuged at 1,000 × g for 15 min. Plasma was aliquoted and stored at − 80 °C until RNA extraction.

### HIV *pol* gene amplification and resistance analysis

Viral RNA was extracted using the High Pure Viral RNA Kit (Roche, Cat. 11858882001), and cDNA was synthesized with PrimeScript II 1st Strand cDNA Synthesis Kit (TaKaRa, Cat. 6210 A). The HIV *pol* gene (protease/reverse transcriptase/integrase) was amplified by nested PCR using TransTaq DNA Polymerase High Fidelity Kit (TransGen, Cat. AP131-13).

Bidirectional Sanger sequencing was performed, and the raw sequences were assembled, edited, and aligned using DNASTAR Lasergene v17. The consensus sequences were then analyzed with the Stanford HIVdb v9.0 to identify resistance mutations. Resistance levels were classified as sensitive, potential resistance, low-level resistance, intermediate resistance, and high-level resistance. Cases with low-to-high resistance were classified as resistant.

### Statistical analysis

Data were managed in Microsoft Excel 2019 and analyzed using SPSS Statistics v26.0 (IBM Corp.). Continuous and categorical variables were compared using independent t-tests/ANOVA and χ² tests, respectively. A two-tailed *P*-value < 0.05 indicated statistical significance.

## Results

### Demographic characteristics of the participants

HIV *pol* gene fragments were successfully amplified from 108 treatment-naïve individuals. Participants were predominantly male (84.3%), aged > 50 years (54.6%), married (74.1%), and had middle school education (76.8%). Heterosexual contact was the predominant transmission route (88.0%) (Table 1).

**Table 1:** Demographic characteristics and drug resistance rates among newly reported HIV-infected individuals in ganzhou, China

Characteristic	Total *n*(%)	Resistant *n*(%)	Non-resistant *n*(%)	χ²	*P*-value
Gender				1.187	0.587
Male	91 (84.3)	6 (6.6)	85 (93.4)		
Female	17 (15.7)	0 (0)	17 (100.0)		
Age (years)				0.675	0.714
< 30	8 (7.4)	0 (0)	8 (100.0)		
30–50	41 (38.0)	2 (4.9)	39 (95.1)		
> 50	59 (54.6)	4 (6.8)	55 (93.2)		
Marital status				2.779	0.427
Unmarried	19 (17.6)	0 (0)	19 (100.0)		
Married	80 (74.1)	5 (6.3)	75 (93.8)		
Divorced/Widowed	6 (5.6)	1 (16.7)	5 (83.3)		
Unknown	3 (2.8)	0 (0)	3 (100.0)		
Education level				1.666	0.645
Primary or below	35 (32.4)	1 (2.9)	34 (97.1)		
Middle school	48 (44.4)	4 (8.3)	44 (91.7)		
High school or above	8 (7.4)	0 (0)	8 (100.0)		
Unknown	17 (15.7)	1 (5.9)	16 (94.1)		
Transmission route				0.869	0.647
Heterosexual	95 (88.0)	6 (6.3)	89 (93.7)		
Homosexual	10 (9.3)	0 (0)	10 (100.0)		
Unknown	3 (2.8)	0 (0)	3 (100.0)		

### Distribution of drug resistance types

Drug resistance analysis was carried out on 108 *pol* fragments. The results showed that seven sequences showed low-, intermediate-, or high-level resistance to at least one type of drug among NNRTIs, NRTIs, or INSTIs, with a total drug resistance rate of 6.5% (Table 1). Among them, five sequences (4.6%) were resistant to NNRTIs, one sequence (0.9%) was resistant to NRTIs, and one sequence (0.9%) was resistant to INSTIs. No protease inhibitor resistance was detected. Resistance prevalence did not significantly differ by gender, age, marital status, education, or transmission route (all *P* > 0.05) (Table 1).

### Drug resistance mutations and drug resistance characteristics

The results of drug resistance analysis showed that a total of nine mutations were detected among the seven infected individuals with drug-resistant strains (Table 2). Among them, the K103N mutation led to high-level resistance to EFV and nevirapine (NVP) among NNRTI drugs; the Y181C mutation led to high-level resistance to NVP and intermediate resistance to EFV, etravirine, and rilpivirine; the A98G and V179E mutations led to intermediate resistance to dapivirine and NVP and low-level resistance to EFV, etravirine, and rilpivirine; the V108I and E138K mutations respectively led to potential low-level or low-level resistance to the corresponding drugs. In addition, the K70Q mutation related to NRTI drugs was found in one infected individual, leading to potential low-level resistance to emtricitabine and 3TC, and low-level resistance to abacavir, stavudine, didanosine, and TDF. In another infected individual, the main mutation E138K related to INSTIs and the accessory mutation E157Q were identified, leading to potential low-level resistance to bictegravir and DTG, and low-level resistance to cabotegravir, elvitegravir, and raltegravir (Table 2).

**Table 2:** Drug resistance characteristics of newly reported HIV-infected individuals in ganzhou, China

Patient ID	Mutation(s)	Resistance Class	Potential-Low-level Resistance	Low-level Resistance	Intermediate Resistance	High-level Resistance
GZ012	Y181C	NNRTI	-	-	EFV, ETR, RPV	NVP
GZ139	K70Q	NRTI	FTC, 3TC	ABC, D4T, DDI, TDF	-	-
GZ348	V108I	NNRTI	DOR, EFV	NVP	-	-
GZ393	A98G, V179E	NNRTI	DOR	EFV, ETR, RPV	DPV, NVP	-
GZ566	E138A	NNRTI	ETR	RPV	-	-
GZ750	K103N	NNRTI	-	-	-	EFV, NVP
GZ770	E138K, E157Q	INSTI	BIC, DTG	CAB, EVG, RAL	-	-

INSTI: Integrase strand transfer inhibitor; NNRTI: Non-nucleoside reverse transcriptase inhibitor; NRTI: Nucleoside reverse transcriptase inhibitor; 3TC: Lamivudine; ABC: Abacavir; BIC: Bictegravir; CAB: Cabotegravir; D4T: Stavudine; DDI: Didanosine; DOR: Doravirine; DPV: Dapivirine; DTG: Dolutegravir; EFV: Efavirenz; ETR: Etravirine; EVG: Elvitegravir; FTC: Emtricitabine; NVP: Nevirapine; RAL: Raltegravir; RPV: Rilpivirine; TDF: Tenofovir disoproxil fumarate

## Discussion

According to the data reported by the Health Commission of Ganzhou, as of December 31, 2022, a total of 4,760 cumulative HIV/AIDS cases had been reported in Ganzhou, with 3,623 surviving cases. Although the absolute number of HIV/AIDS cases in Ganzhou is relatively small, the number of new HIV diagnoses has increased rapidly in recent years, especially among people aged 50 and above, showing an increasing trend year by year [11]. The results of this study showed that the HIV-infected individuals in Ganzhou are mainly male (84.3%), middle-aged and elderly people over 50 years old (54.6%), and those with a low educational level (junior high school education or below, 76.8%), and heterosexual transmission accounts for 88.0%. These findings further enrich the epidemiological data on HIV in Ganzhou and provide an evidence-based foundation for local epidemic prevention and control efforts.

Research on HIV drug resistance in Jiangxi Province remains limited. Zhang et al. conducted drug resistance analyses among ART-naïve HIV/AIDS patients in the province in 2013 and 2015, with sample sizes of 34 and 112 cases respectively, and found that the reported drug resistance mutation rates were 0% and 4.46% respectively [12, 13]. This study found a 6.5% prevalence of PDR among newly diagnosed HIV-infected individuals in Ganzhou. While significantly lower than the 10–15% reported in global high-prevalence regions, this rate falls within China’s moderately low range (4.0–30.1%) and exceeds the WHO’s 5% threshold for low-level resistance warning [14, 15]. These findings indicate an increasing trend of PDR among newly diagnosed HIV-infected individuals in Jiangxi Province and underscore the urgent need for expanded resistance surveillance among high-risk groups, such as men who have sex with men and people aged 50 years and above.

China’s free ART program, initiated in 2003, has heavily relied heavily on EFV-based regimens for two decades. The present study found that NNRTIs was predominant (4.6% vs. 0.9% for NRTIs/ INSTIs), which reflects this historical usage pattern. In particular, mutations such as A98G, K103N, V179E, and Y181C confer resistance to traditional NNRTIs (EFV/ NVP) [16, 17]. Notably, these findings come at a time when China is transitioning to INSTI-based regimens (DTG, raltegravir) and newer NNRTIs (doravirine, anvirolen) in accordance with the 2024 Guidelines, which may change future resistance profiles. The absence of major protease inhibitor resistance aligns with their limited use as alternative regimens (darunavir/cobicistat, lopinavir/ritonavir). These data provide crucial baseline resistance information during this treatment paradigm shift and support the guideline in China - recommended avoidance of EFV/NVP in regions with established NNRTI resistance.

Studies have demonstrated that the TDF + 3TC-based NRTI regimen possesses a high genetic barrier to resistance, as it requires the accumulation of both M184V and K65R mutations to develop clinical resistance [18, 19]. China initiated the implementation of this treatment protocol in 2016. As of 2023, the prevalence of transmitted drug resistance to NRTIs in China has remained stable at a low level (1.3%) [6]. The current study revealed that single mutations (e.g., K70Q) only confer potential low-level or low-level resistance, further validating the regimen’s ability to maintain low resistance rates in clinical practice. These findings indicate that the TDF + 3TC + EFV regimen remains a viable first-line option for China’s free treatment program.

DTG is a second-generation INSTI with a high resistance barrier and excellent efficacy. Since its global launch in 2013, DTG has become a core first-line drug recommended by the World Health Organization. In 2021, the Chinese AIDS treatment guidelines also recommended the DTG + 3TC dual regimen as the preferred first-line treatment for newly diagnosed patients. This study detected one case of INSTI PDR with resistance mutations E138K/E157Q. Notably, these mutations confer potential low-level resistance to DTG. Although the DTG resistance mutation identified in this study does not currently meet the clinical resistance threshold, a number of studies indicated that HIV-infected individuals with potential resistance not only had a higher proportion of resistance, but also had a higher rate of virological failure when receiving first-line treatment [20]. These findings call for enhanced clinical surveillance regarding potential drug resistance and associated transmission risks in China.

This study has the following two limitations: Firstly, the modest sample size and low resistance prevalence limited risk factor analysis for specific mutations; Secondly, non-random sampling may affect generalizability. Nevertheless, as Ganzhou’s inaugural HIV resistance study, our work provides valuable baseline data for local epidemic control.

In summary, a drug resistance study was conducted among newly diagnosed HIV-infected individuals in Ganzhou. Results showed that the HIV PDR among them was 6.4%, mainly to NNRTIs, and drug-resistant strains resistant to INSTIs have emerged. Sustained PDR surveillance is crucial to reduce the spread of drug-resistant strains and to provide a reference for optimizing the treatment regimen.

## Data Availability

The datasets used and analyzed during this study are available from the corresponding author on reasonable request.

## Abbreviations

3TC: Lamivudine
ART: Antiretroviral therapy
DTG: Dolutegravir
EFV: Efavirenz
HIV: Human immunodeficiency virus
INSTIs: Integrase strand transfer inhibitors
NNRTIs: Non-nucleoside reverse transcriptase inhibitors
NRTIs: Nucleoside reverse transcriptase inhibitors
NVP: Nevirapine
PDR: Primary drug resistance
TDF: Tenofovir disoproxil fumarate
